# Health anxiety and healthcare costs in Japanese individuals: an Internet survey

**DOI:** 10.1080/21642850.2014.945935

**Published:** 2014-08-20

**Authors:** Osamu Kobori, Mayuko Okita, Tetsuya Shiraishi, Tadashi Hasegawa, Masaomi Iyo

**Affiliations:** ^a^ Centre for Forensic Mental Health, Chiba University, 1-6-1-3-1202, Utase, Mihamaku, Chiba2608670, Japan; ^b^ Department of Psychiatry, Chiba University, Chiba, Japan

**Keywords:** Internet-based research, mental health and disorder, health anxiety, hypochondriasis, healthcare cost

## Abstract

*Aim*: Health anxiety, also known as hypochondriasis, is classifiable as an anxiety disorder. The aim of this study was to examine the relationship between health anxiety and healthcare costs. *Method*: Participants – 100 Japanese individuals from the general population with chronic health problems and 100 without chronic health problems – were recruited via the Internet. They completed self-report scales measuring health anxiety, state anxiety, depression, obsessionality, and a scale specifically developed for this study that measured the use of healthcare services and the personal costs of respondents' healthcare. *Results*: Health anxiety was associated with more incidents of inpatient care and greater healthcare expenditure. These associations remained significant even after controlling for state anxiety, depression, obsessionality, and the presence of chronic health problems. *Conclusion*: We conclude that health anxiety is related to personal as well as social costs in Japan.

## 1. Introduction

### 1.1. The cognitive-behavioural perspective of health anxiety

Hypochondriasis may be better categorised as an anxiety disorder (health anxiety) than a somatoform disorder as it has much in common with both obsessive–compulsive disorder (OCD) and panic disorder (Salkovskis & Warwick, 1986; Warwick & Salkovskis, 1990). The cognitive-behavioural hypothesis of health anxiety proposes that people who experience severe and persistent health anxiety have a relatively enduring tendency to misinterpret bodily symptoms, bodily variations, medical information, and any other health-related information as evidence that they currently have, or are at risk of having, a serious physical illness. Thus, health anxiety is said to occur as a result of catastrophic misinterpretations of health-related information.

Safety-seeking behaviour is an important and counterproductive feature of health anxiety, as it is in other anxiety disorders. Health anxiety sufferers often attempt to cope with their anxiety by carrying out safety-seeking behaviours including avoidance of hospitals, specialist clinics, sick people, blood, or the mentally ill; repeated medical consultations and tests; self-checking of their body, memory, or vision; compulsive requests for reassurance; and repeated searches for reassuring information on websites (Muse, McManus, Leung, Meghreblian, & Williams, 2012). Such behaviours are, of course, usually directed at attempts to ensure that the threat of having and/or developing a serious illness does not affect them or others, and that they are not responsible for such threats. However, these safety-seeking behaviours may help maintain health anxiety by preventing the acquisition of information that disconfirms inaccurate beliefs through a misattribution of safety and/or by diverting attentional resources away from disconfirming information.

### 1.2. Health anxiety and healthcare cost

Health anxiety not only causes great suffering for patients and those around them but also is costly in terms of greater use of medical care utilisation. For example, individuals suffering from health anxiety desperately seek to identify the physical causes of their symptoms and will often consult several medical professionals. Evidence suggests that social costs of health anxiety are high. In undergraduates, health anxiety is linked to increased doctor visits, decreased academic performance, and co-occurring psychological distress (Abramowitz, Deacon, & Valentiner, 2007). Patients with somatisation disorder incur healthcare costs that are 6–14 times the US national average (Smith, Monson, & Ray, 1986). Furthermore, higher numbers of attendances by doctors have been noted in those with high health anxiety (Seivewright et al., 2004), and evidence suggests that this high service use persists over the long term (Reid, Crayford, Patel, Wessley, & Hotopf, 2003). Barsky, Ettner, Horsky, and Bates (2001) found that individuals who reported elevated health anxiety were more inclined to seek health reassurance through both general and specialty health consultations and had more hospital admissions and emergency ward visits. A separate study that examined healthcare costs found that patients with severe health anxiety had approximately twice the annual healthcare costs when compared to individuals with minimal health anxiety (Barsky, Orav, & Bates, 2005). On the other hand, Barrett et al. (2012) failed to identify an association between severity of health anxiety and social costs among 444 people with high health anxiety. Barrett et al. (2012) noted that service-use data were collected retrospectively by self-report; therefore, the reported costs may not be completely accurate. It should be noted that the self-report measure they used asked participants to recall contacts with all health and social care services for the six months preceding the study. Thus, it might have been difficult for the responders to accurately remember all the services used because of the relatively long target period.

Hypochondriasis is associated with considerable personal cost. Given that individuals with health anxiety are preoccupied with their health status, they might spend money, time, and effort on their healthcare; for example, they may choose to have acupuncture for chronic back pain or purchase over-the-counter drugs for inexplicable headaches. Thus, it is vital to assess health anxiety in terms of unnecessary healthcare utilisation, reduced quality of life, and increased personal healthcare costs (Bleichhardt & Hiller, 2007; Fink, Ørnbøl, & Christensen, 2010). In addition, cultural factors, such as a country's healthcare structure, may affect reassurance seeking and related social and personal costs. For example, in Japan, citizens pay 30% of both outpatient and inpatient treatment costs and the remainder is covered under the public national insurance system. One of the problems of this system, from the government's point of view, is that citizens can go to any hospital or clinic, even without referrals. Thus, people can seek medical consultations in clinics outside the area in which they live, which may facilitate ‘doctor-shopping’. This may be one of the reasons why Japanese citizens seek and receive medical consultations almost twice as frequently as Europeans do. The average number of medical consultations per year was 13.4 in Japan, while this number was 5.9 in Britain, 7.8 in Germany, and 6.9 in France (Organisation for Economic Cooperation and Development, 2012).

### 1.3. Purpose of this study

This study examines the relationship between health anxiety and healthcare costs of Japanese individuals. In particular, this study tested the following hypotheses:

1. Regardless of health problems, higher health anxiety would be associated with greater medical care utilisation, even after controlling for general psychopathology.
2. Regardless of health problems, higher health anxiety would be associated with greater expenditure on healthcare, even after controlling for general psychopathology.

## 2. Method

### 2.1. Design

This study employed an Internet survey, which enabled us to recruit participants from a variety of regions (i.e. urban and local areas) and age groups.

The participants were recruited via Rakuten Research, an online marketing research company that holds approximately 2.3 million Japanese enrolments. Respondents aged 18–65 years were contacted by email and asked to take part in this study. If they agreed, they indicated whether they had chronic health problems before completing the rest of the questionnaires. In this study, chronic health problems were defined as health conditions that required continual treatment for more than three months prior to the study. Recruitment continued until the number of individuals with and without chronic health problems reached 100.

### 2.2. Participants

Participants were 200 Japanese individuals from the general population (113 females and 97 males), with a mean age of 43.85 years (SD = 10.025). On the basis of their self-reports of chronic health problems, they were assigned to either a chronic or a non-chronic group.

### 2.3. Measures

#### 2.3.1. Short Health Anxiety Inventory

The Short Health Anxiety Inventory (SHAI) (Salkovskis, Rimes, Warwick, & Clark, 2002) is a self-report measure that contains 18 items assessing health anxiety independently of physical health status. Using a multiple-choice format, items measure worry about health, awareness of bodily sensations or changes, and feared consequences of having an illness. The Japanese version of the SHAI was developed by Yamauchi, Matsuoka, Himachi, Sasagawa, and Sakano (2009) and demonstrates sufficiently high internal consistency and convergent and discriminant validity.

#### 2.3.2. Obsessive–Compulsive-Inventory

The Obsessive–Compulsive-Inventory (OCI) (Foa, Kozak, Salkovskis, Coles, & Amir, 1998) consists of 42 items in seven subscales: Washing, Checking, Doubting, Ordering, Obsessing, Hoarding, and Mental Neutralising. The Japanese version was developed by Ishikawa, Kobori, Nagaoka, and Shimizu (Unpublished results).

#### 2.3.3. The State-Trait Anxiety Inventory

The State-Trait Anxiety Inventory (STAI) (Spielberger, Gorsuch, & Lushene, 1970) is made up of two scales: ‘State’ anxiety (STAI-S) and ‘Trait’ anxiety (STAI-T). Twenty items, each with four response options, comprise each scale. State anxiety items address respondents' current anxiety, whereas trait anxiety items are designed to assess self-reported typical anxiety. This study employed the Japanese version of the STAI-S (Nakazato & Mizuguchi, 1982).

#### 2.3.4. Center for Epidemiologic Studies Depression Scale

The Center for Epidemiologic Studies Depression Scale (CES-D) (Radloff, 1977) consists of 20 items rated on a 4-point scale ranging from 0 (*never or few*) to 3 (*usually*). The symptom score is the sum of the 20 items. The Japanese version of the CES-D was found to be applicable to Japanese adults in the community (Shima, Shikano, & Kitamura, 1985).

#### 2.3.5. Healthcare cost

Participants' healthcare cost was measured with four items developed specifically for this study:

- *Service utilisation*: Participants were asked how many times over the past month they had visited a healthcare service, including clinics, hospitals, and public healthcare centres. Responses were scored with a 5-point Likert scale: (1) never, (2) once, (3) 2–4 times, (4) 5–10 times, and (5) more than 11 times.
- *Inpatient care*: Participants were asked how many days they had stayed in the hospital during the past month. Responses were scored with a 5-point Likert scale: (1) never, (2) 1 day, (3) 2–4 days, (4) 5–10 days, and (5) more than 11 days.
- *Trips by ambulance*: Participants were asked how many times during the past month they had called an ambulance or asked someone to call an ambulance for them. Responses were scored with a 5-point Likert scale: (1) never, (2) once, (3) 2–4 times, (4) 5–10 times, and (5) more than 11 times.
- *Personal expenditures*: Participants were asked to indicate how much they spent over the past month on their own healthcare (e.g. doctor's visit, medical examinations, prescribed drugs, over-the-counter drugs, alternative therapy, or private medical insurance). Participants filled in the total sum in units of 1000 yen (approximately 10 USD).

### 2.4. Analysis plan

Hierarchical regression analyses were employed to test whether health anxiety would predict service utilisation, inpatient care, trips by ambulance, and personal expenditure, after controlling for demographics (age, gender, and presence of chronic health problems) and general psychopathology (anxiety, depression, and OCD symptoms). Logistic regression analyses with the block-entry model were employed when the dependent variables were not normally distributed. Data were analysed by using statistical package for the social sciences version 19.0, and the significance level was defined as *p* < .05.

## 3. Results

### 3.1. General psychopathology

Table 1 presents the means and standard deviations of each scale for the chronic and non-chronic group. A one-way analysis of variance revealed that the chronic group scored significantly higher than the non-chronic group on most of the variables; however, the differences were only marginally significant for inpatient care and trips by ambulance.

**Table 1.** Differences in scales between individuals with and without chronic health conditions.

	Alpha	Total		Chronic group		Non-chronic group			
		Mean	SD	Mean	SD	Mean	SD	*F*(1, 198)	*p*
HAI	0.926	16.070	9.388	18.930	10.554	13.210	7.018	20.368	.000
OCI	0.983	17.110	27.569	21.750	32.607	12.470	20.520	5.802	.017
STAI-S	0.691	45.735	11.797	47.610	11.968	43.860	11.375	5.158	.024
CES-D	0.864	36.220	11.605	38.330	12.244	34.110	10.573	6.804	.010
Service utilisation		2.140	1.032	2.600	0.932	1.680	0.920	49.360	.000
Inpatient care		1.105	0.605	1.180	0.796	1.030	0.300	3.108	.079
Ambulatory visit		1.080	0.505	1.140	0.697	1.020	0.141	2.851	.093
Personal expenditure		12.555	15.401	14.990	16.129	10.120	14.307	5.102	.025

Notes: SHAI, Short Health Anxiety Inventory; OCI, Obsessive–Compulsive-Inventory; STAI-S, State-Trait Anxiety Inventory-State Scale; CES-D, Center for Epidemiologic Studies Depression Scale; SD, Standard Deviation.

### 3.2. Regression analysis

Tables 2 and 3 present the results of the regression analyses. Because most participants selected ‘none’ or ‘never’ for inpatient care and trips by ambulance, these dependent variables were collapsed into ‘never’ or ‘more than once’ or ‘more than 1 day’ for the logistic regression analysis. Moreover, because all those who had called the ambulance were female, gender was removed from independent variables for the analysis of trips by ambulance.

**Table 2.** Hierarchical regression analysis of service utilisation and personal expenditure predicted by demographics, general psychopathology, and health anxiety.

		Service utilisation			Personal expenditure		
		*R*^2^	*F* change	*β*	*R*^2^	*F* change	*β*
Step 1	Gender	0.201	16.404***	.038	0.040	2.750*	−.083
	Age			.046			.089
	Group			−.409***			.068
Step 2	OCI	0.211	0.845	.089	0.060	1.308	−.304**
	STAI-S			−.040			−.057
	CES-D			−.051			.034
Step 3	SHAI	0.219	1.950	.125	0.143	18.688***	.406**

Notes: SHAI, Short Health Anxiety Inventory; OCI, Obsessive–Compulsive-Inventory; STAI-S, State-Trait Anxiety Inventory-State Scale; CES-D, Center for Epidemiologic Studies Depression Scale.

*p < .05.

**p < .01.

***p < .001.

**Table 3.** Logistic regression analysis of impatient care and trips by ambulance predicted by demographics, general psychopathology, and health anxiety with the block-entry model.

	Inpatient care					Trips by ambulance				
	Wald	df	Sig.	OR	95% CI	Wald	df	Sig.	OR	95% CI
Gender	−1.601	1	.109	0.170	0.019–1.487	–	–	–	–	–
Age	−0.994	1	.320	0.949	0.857–1.051	−1.270	1	.204	0.968	0.921–1.017
Group	1.125	1	.261	3.106	0.431–22.354	0.635	1	.525	1.876	0.269–13.053
OCI	−1.925	1	.054	0.940	0.882–1.001	−0.736	1	.462	0.984	0.944–1.027
STAI-S	−0.872	1	.383	0.939	0.816–1.081	−0.459	1	.646	0.976	0.880–1.082
CES-D	1.481	1	.139	1.144	0.957–1.368	0.402	1	.688	1.033	0.881–1.213
SHAI	1.955	1	.051	1.114	1.000–1.242	1.524	1	.127	1.077	0.979–1.185

Notes: SHAI, Short Health Anxiety Inventory; OCI, Obsessive–Compulsive-Inventory; STAI-S, State-Trait Anxiety Inventory-State Scale; CES-D, Centre for Epidemiologic Studies Depression Scale; OR, Odds Ratio.

After adjusting for demographics and general psychopathology, the SHAI significantly predicted personal expenditure and marginally predicted inpatient care. However, SHAI did not predict service utilisation or trips by ambulance. In terms of other variables entered in this model, the OCI negatively and significantly predicted personal expenditure, and negatively and marginally predicted inpatient care, which may reflect a miserly spending style related to obsessional tendencies.

## 4. Discussion

The purpose of this study was to examine the relationship between health anxiety and healthcare costs in Japanese individuals. The analysis of the group differences revealed that individuals with chronic health conditions not only were more anxious about their health, but also utilised healthcare services and spent more money on healthcare. This is consistent with the notion that actual physical illness may be a major vulnerability factor in promoting health anxiety, a finding for which there is evidence from general medical settings (Kellner, 1985).

The results of the regression analyses suggest that even after adjusting for demographics and general psychopathology, health anxiety was found to be associated with the number of days in inpatient care and personal healthcare expenditure. This result suggests that health anxiety is costly for society because the government has to cover 70% of the cost for inpatient care in Japan. Moreover, health anxiety can be costly for individuals; those with greater health anxiety spend more money not only on medical consultations, examinations, and prescriptions, but also on over-the-counter drugs, alternative therapies, and private medical insurance. On the other hand, health anxiety was not associated with service utilisation and trips by ambulance, suggesting that individuals with health anxiety did not visit healthcare services, including clinics, hospitals, and public healthcare centres, more often than those without health anxiety. Individuals with health anxiety may try to take care of themselves by purchasing healthcare products rather than frequently visiting doctors, especially when they are aware of the ‘cost’ of such frequent visiting, which may include consultation fees and bothering busy doctors.

Such costly behaviours are considered safety-seeking behaviours, which in turn intensify and maintain health anxiety. First, safety-seeking behaviours prevent the acquisition of information that disconfirms inaccurate threat beliefs through misattribution and/or by diverting attentional resources away from information leading to disconfirmation. Spending money on healthcare may lead individuals to believe that they are doing their best to stay healthy, and they may feel that such behaviours prevent the development or exacerbation of serious illness. Second, engaging in these behaviours can be misinterpreted as evidence that they currently have, or are at risk of having, a serious physical illness. Thus, the assessment and treatment of health anxiety should focus on the ways individuals spend money on their healthcare and other safety-seeking behaviours such as avoidance, repeated medical consultations and examinations, self-checking, strict dieting, and repeated searches for reassuring information on websites.

## 5. Limitations and future directions

This research has some limitations, and many important issues remain open to empirical investigation. First, the sample size was relatively small and limited to a Japanese population. Further research should be conducted with larger groups and more diverse populations. Second, the participants were classified into groups with and without chronic health conditions on the basis of their self-report, which limits the reliability of diagnoses. Future research should employ more standardised screening, and the target of chronic health conditions should be limited to a specific area of illness, such as respiratory dysfunctions or gastrointestinal disease.

Administration of psychological questionnaires via the Internet has gained popularity in recent years and has many advantages. However, before questionnaires that were originally developed as paper-and-pencil measures can be confidently administered over the Internet, it is necessary to document the equivalence of the paper and computer-generated versions, although the psychometric properties of the online version of some instruments, such as the OCI, have been confirmed (Coles, Cook, & Blake, 2007).

In Japan, people can go to any hospital or clinic, even without referrals. Consequently, their medical consultations are not restricted to the clinics located in the area in which they live, and this might facilitate ‘doctor-shopping’. From service providers' point of view, repeated unnecessary consultations are often a nuisance for primary care physicians, but even such consultations can be profitable for their clinics and, therefore, rewarding for physicians. These characteristics of the Japanese healthcare system limit the generalisability of this study results.
